# Adaptive cruise control for electric vehicles using hybrid-mode MPC

**DOI:** 10.1038/s41598-026-51908-x

**Published:** 2026-06-05

**Authors:** Ahmed E. Sharkawy, Ahmed M. Ali, Mostafa Sh. Asfoor, Mostafa I. Yacoub

**Affiliations:** Automotive Engineering Department, Military Technical College, I. Fangari, Cairo, 11766 Egypt

**Keywords:** Electric vehicle, Advanced driver assistance systems, Adaptive cruise control, Dynamometer testing, Model predictive controller, System identification, Parameter estimation, Energy science and technology, Engineering, Mathematics and computing

## Abstract

The rapid transition towards electromobility urges a concurrent focus on safety and intelligent vehicle control. In this regard, Advanced Driver Assistance Systems (ADAS) are paramount, playing a critical role in mitigating human error and enhancing road and passenger safety. However, challenges remain in the robust formulation and integration of such control systems, particularly considering the difficulty in modeling sophisticated architectures and the power synergy paths of hybrid/electric drivelines. This paper presents a comprehensive, novel methodology that utilizes a single-platform solution for model parameters tuning and online optimization of the control layers within an Adaptive Cruise Control (ACC) system for Electric Vehicles (EVs). To this aim, an intelligent Model Predictive Control (MPC) is developed, based on decentralized control modes for cruising, spacing, and braking. A unified prediction model is implemented to provide look-ahead estimation of the driving situation based on real-time measurements. The efficacy of the model prediction and control mode swapping was investigated through experimental testing of a real EV on a chassis dynamometer, with an emulated lead vehicle detected by an on-board LiDAR sensor. The single-platform, featuring updated model parameters and optimized control gains, demonstrated an ability to maintain speed-tracing and precise spacing when exposed to different disruptive scenarios. The per-mode tracking accuracy achieved 98% in cruise control, 87.8% in spacing control, and 55.0% in braking mode under coasting-only constraints. The proposed work thus offers a significant, unified solution to handle the complex challenges of driveline modeling and control system design, mitigating computational and technical difficulties.

## Introduction

Electric vehicles (EVs) contribute to the improvement of urban transportation systems due to their lower carbon footprint and increasingly challenging performance. Increasing efforts promoting road- and passengers’ safety have shed light on the significance of intelligent advanced driver assistance systems (ADAS) to raise the awareness and response of drivers in potentially hazardous situations^1^.

The development of different driver assistance algorithms depends in first place on accurate and precise modeling of the powertrain and entailed vehicle dynamics. In this regard, precise estimation of electric vehicles’ drivetrain parameters is an important consideration to yield a high degree of confidence in control decision^2^.

The challenge of accurate powertrain modeling and online update of estimated parameters has grasped the attention of many researchers as summarized in Table 1. A significant milestone in this regards is the suitability of such adaptive models to be integrated to sophisticated ADAS systems conducting or advising cruising and lane-change decisions in real-time^3,4^.

The simultaneous use of experimental measurements during driving cycle testing to iteratively estimate and correct vehicle parameters is a typical approach that has been considered in multiple works. In^5^, the testing data has been implemented sequentially to estimate, evaluate, and validate crucial modeling parameters of the batteries and electric motors. This contributes to applying a verified model-based control algorithm, such as the car-following control proposed in^6^, and the work proposed in^7^ developed a control algorithm that uses Signal and Phase Timing information from actuated traffic lights to optimize vehicle speed and save energy at traffic intersections, via a model predictive controller (MPC).

The studies^8^ and^9^ used maximum likelihood and Bayesian optimization methods to estimate and validate the battery State of Charge (SOC) and tire modeling parameters, respectively. These methods demonstrate high robustness and estimation accuracy, yet with reduced effectiveness under highly nonlinear systems. This enhances the performance of the developed control algorithms, especially those relying on accurate system state estimation, as demonstrated in^9,10^.

The gradient-based methodologies in^11^ and^12^ have been used to determine vehicle side-slip angle with adhesion coefficient and battery State-of-Health (SOH), respectively. The achieved results in both works proved the ability of gradient-based approaches to yield fast convergence with minimal computational complexity. This established advanced control techniques, such as the gradient-based augmented Lagrangian MPC introduced in^13^ for trajectory tracking control.

Evolutionary algorithms (EAs) combined with the nonlinear least-squares method have been most frequently used to better explore the search space for parameters estimation^14–17^. While EAs demonstrated significant fulfillment of the estimation features, they require long training times^18^. Nevertheless, their computational benefits led to enhanced control techniques, such as the rule-based control proposed in^19^, for optimally determining a hybrid EV’s power split.

**Table 1:** Comparative analysis of control-oriented modeling parameters estimation approaches.

Category	Estimation Method	Features and Limitations Aspects	Related Control Algorithms	Representative Application
**Statistical approaches**	Maximum likelihood	Precise large-scale estimation, but computationally complex and stuck in local optima^9,20^.	Sensor fault-tolerant control for EV induction motors^20^.	Uncertainty mitigation in online SOC estimation of lithium-ion batteries^9^.
	Generalized method of moments	Interpretable optimal estimates, but complex computations and datasets are required^21,22^.	Nonlinear MPC enhances the yaw motion stability of an EV considering safety constraints^21^.	Obstacle avoidance control using a developed MPC under uncertainty conditions^22^.
	Bayesian estimation	Handling non-linearity, but vulnerable to local optima entrapment^10^.	Car-following controllers using both linear control and MPC^23^.	Robust commercial ACC experimentally validated using field data^23^..
**Gradient-based methods**	Gradient descent	Rapid convergence with accurate estimates, but limited nonlinearity handling^11^.	Gradient-based augmented Lagrangian MPC with processor-in-loop validation^13^.	Predictive trajectory tracking control in different driving scenarios with low computational cost^13^.
	Conjugate gradient	Computationally efficient with local optima avoidance, but limited nonquadratic problem handling^24^.	An optimal control algorithm based on a well-defined optimization problem^24^.	Experimentally validated SOH modeling parameters are integrated into battery management systems^12^.
	Newton’s method	High convergence rate, but with high computational cost^25^.	Iterative learning control based on linear models^25^.	Accelerated wheel force file generation for vehicle testing^25^.
**Evolutionary algorithms**	Genetic algorithm	Precise estimates of nonlinear systems; slow convergence^18^.	Rule-based power split control strategy for a hybrid EV^19^.	Battery state of power estimation for EV energy management system^18^.
	Particle swarm optimization	Global search capability in large-scale problems, but with high computational cost^26^.	Adaptive fuzzy controller optimizes torque allocation via a multi-objective problem^27^.	Implementation of real-time energy management strategy for plug-in hybrid electric buses^27^..
	Simulated annealing	Accurate estimates with robust local optima avoidance, but with slow convergence speed^28^.	Rule-based control implemented through optimized parameters and predefined thresholds^19^..	Accurate identification of the permanent magnet synchronous motor modeling parameters^28^.
**Non-linear least square**	Levenberg-Marquardt	Accurate estimation for highly nonlinear systems, but with slow convergence and limited scalability^14^.	Artificial Neural Network (ANN) controller using Levenberg-Marquardt algorithm^15^.	Traffic control of intelligent vehicles by predicting traffic patterns and optimizing travel paths^14^.
	Gauss-Newton	Fast and accurate estimation for large-scale problems, but vulnerable to trapping in local minima^15^.	ANN and Proportional Integral Derivative (PID) controllers with simplified system orders^15^.	Control and identification of an electric power steering system for autonomous vehicles^15^.
	Trust region reflective algorithm	Robust global convergence for large-scale problems, but exhibiting high computational demands^16^.	Deep reinforcement learning with trust region optimization for vehicular trajectory planning^17^.	Traffic evacuation planning and management during hazardous situations^29^.

Model-based control approaches are typically advantageous when high-fidelity models are integrated to vehicular control systems. Such control schemes proved a significant potential to yield desired results, thanks to attainable accurate estimation. In this context, MPC has been widely implemented in adaptive cruise control systems (ACC), including the collision avoidance, traffic flow regulation, and fuel saving objectives^30,31^. The extent of using validated models within different control schemes is also identifiable in fuzzy-logic control^27^ and evolutionary algorithms^15^. In^19^, a simplified rule-based approach has been introduced for steering system identification based on simulated annealing for parameter estimation.

Dynamic Programming (DP) provides a theoretical global optimum but requires complete a priori knowledge of the entire driving cycle, which inhibits its real-time embedded deployment. Recent works consistently treat DP as an offline performance benchmark rather than a deployable controller. The work^32^ developed an EV-specific Eco-ACC strategy and showed that the proposed MPC produces a smoother torque output and reduces battery-discharge peaks compared with the DP-based Eco-ACC, thereby extending battery life. The work in^33^reported fuel-economy improvements of 41.23% with nonlinear MPC against 52.25% with DP in a connected car-following scenario.

In light of the investigated previous works, the challenging trade-off between the accuracy, performance, and computational efficiency of vehicular control – with a particular focus on ACC – can be perceived. The analysis of this challenge is categorically presented in Table 1 from the perspectives of estimation methodology, control approach, and the inherent limitation in each work. It can be put forth that the development of MPC architecture to promote the use of high-fidelity models within computationally-efficient schemes is a trending approach that has the potential to yield near-optimal results.

This paper presents a novel integration of a high-fidelity EV model with a hybrid-mode MPC for vehicular ACC. The proposed approach utilizes real-time LiDAR measurements to adaptively select both the reference signals and cost-function weights for each operational mode. Additionally, a LabVIEW-based graphical user interface was designed for simultaneous tuning of model parameters and ACC settings based on acquiring and visualizing experimental measurements. Furthermore, both dynamometer and on-road testing procedures have been conducted to evaluate the proposed approach. The achieved results put forward the ability of the developed hybrid-mode MPC to ensure efficient cruise control tasks with minimal spatial and speed errors.

This paper is organized as follows: Section “Mathematical modeling” introduces longitudinal dynamics modeling of an electric vehicle. Section “Adaptive cruise control using hybrid-mode MPC” explains the ACC system using switched MPC. Section “Experimental testing procedures and offline model-parameter estimation” describes the experimental model validation and the implementation of the proposed ACC based on hybrid-mode MPC. Results analysis of the proposed ACC system and the paper’s conclusion are introduced in Sections “Results analysis” and “Conclusion”, respectively.

## Mathematical modeling

**Fig. 1:**
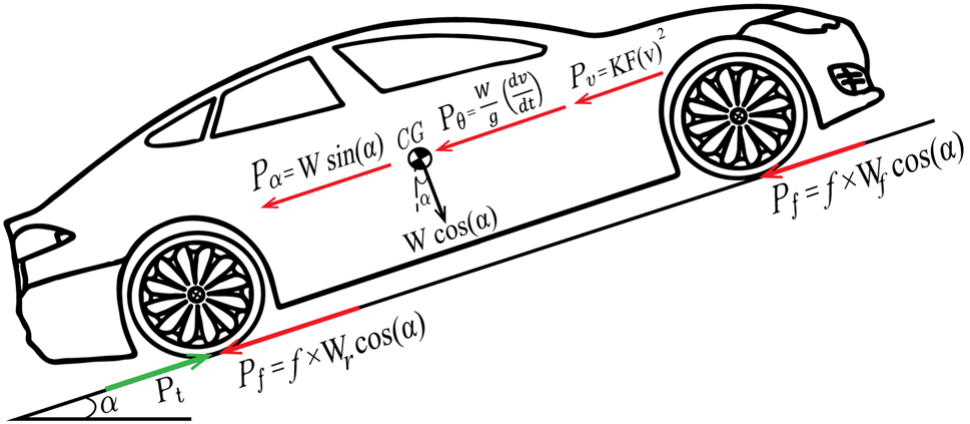
Schematic drawing for longitudinal vehicle dynamics.

The implemented model in this work depicts an experimental vehicle, comprising a pure-electric drive-line with two in-wheel motors and a battery pack. The longitudinal vehicle dynamics is primarily calculated using a backward model as illustrated in Fig. 1 to calculate the required traction effort $(P_{t})$ based on a given driving cycle speed as

1 $$\begin{aligned} P_{t} =&P_{v}+P_{f}+P_{\alpha }+P_{\theta }, \quad \end{aligned}$$

where $(P_{v})$, $(P_{f})$, $(P_{\alpha })$, and $(P_{\theta })$ denote the aerodynamic resistance, the rolling resistance, the grade resistance, and the inertia resistance respectively.

These resistances can be individually calculated as

2 $$\begin{aligned} P_{v} =&KF(v)^2, \end{aligned}$$

3 $$\begin{aligned} P_{f} =&W f\cos (\alpha ), \end{aligned}$$

4 $$\begin{aligned} P_{\alpha } =&W \sin (\alpha ), \quad \text {and} \end{aligned}$$

5 $$\begin{aligned} P_{\theta } =&\frac{W}{g}\left( \frac{dv}{dt}\right) , \end{aligned}$$

where (*v*) denotes vehicle speed, $(\alpha )$ is the road inclination angle, and (*g*) is the gravitational acceleration. The output torque of the electric motors $(T_{mot})$ should overcome the load torque value $(T_{load})$ that can be calculated from equation 6 which can be expressed as

6 $$\begin{aligned} {T_{load}}={P_{t} r_{d}}. \end{aligned}$$

The interdependence between lateral and longitudinal slip ratios of the tire is a crucial input for vehicular cruise control. The semi-empirical model in^34^ is considered to calculate the achievable longitudinal force $(F_{x})$ and tire slip ratio $(\lambda )$ under different vertical forces $(F_z)$ as

7 $$\begin{aligned} F_{x,j} = D \sin [C \arctan (\psi _j)], \quad j = f, r \end{aligned}$$

where the subscript *j* denotes the tire position index (front or rear) and the slip input transformation $(\psi )$ is defined as

8 $$\begin{aligned} \psi _j = B \lambda _j - E(B \lambda _j - \arctan B \lambda _j). \end{aligned}$$

The slip ratio can hence be defined based on(9) as

9 $$\begin{aligned} \lambda _j = \frac{\omega _j r_d - v}{\max (\omega _j r_d, v,\mathrm {\varepsilon })}, \end{aligned}$$

where $(\omega )$ is the angular speed of the wheel and $(\varepsilon )$ is a small positive constant (typically 0.01 m/s) that prevents numerical singularities when both the wheel speed and vehicle speed approach zero^35^. For simplification purposes, the effect of suspension dynamics and lateral slip are precluded considering the light weight EV in this work. The empirical parameters in (7) are hence defined as^34,35^

10 $$\begin{aligned} C&= a_0, \end{aligned}$$

11 $$\begin{aligned} D&= a_1 F_{z,j}^2 + a_2 F_{z,j}, \end{aligned}$$

12 $$\begin{aligned} B&= \frac{a_3 F_{z,j}^2 + a_4 F_{z,j}}{C D e^{a_5 F_{z,j}}}, \quad \text {and} \end{aligned}$$

13 $$\begin{aligned} E&= a_6 F_{z,j}^2 + a_7 F_{z,j} + a_8, \end{aligned}$$

where $a_0$–$a_8$ are arbitrary tunable parameters, that depends on normal tire loads $(F_{z,r})$ and $(F_{z,f})$ as

14 $$\begin{aligned} F_{z,r}&= \frac{Wl_f \cos (\alpha ) + h_{cg} [P_t - W\cos (\alpha )]}{2l} \quad \text {and} \end{aligned}$$

15 $$\begin{aligned} F_{z,f}&= \frac{Wl_r \cos (\alpha ) - h_{cg} [P_t - W\cos (\alpha )]}{2l}. \end{aligned}$$

**Fig. 2:**
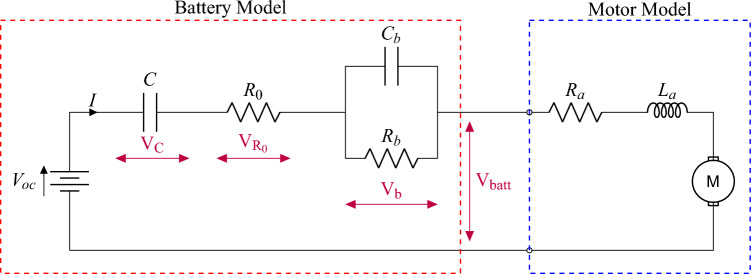
Simplified equivalent circuit model for the vehicle’s electrified propulsion system.

The implemented drive-line model has been conceptually presented in^16^. The model consists of a first-order Thevenin model representing the charging and discharging dynamics of the lithium iron phosphate (LiFePo_4_) batteries empowering two in-wheel permanent magnet DC motors with the equivalent circuit shown in Fig. 2.

The equivalent dynamics of the battery voltage under load can be calculated as

16 $$\begin{aligned} V_{batt}&=V_{oc}-V_{b}-V_{R_0}-V_{C}, \end{aligned}$$

17 $$\begin{aligned} I&= C_b \ \dot{V_b}+\frac{V_b}{R_b},\end{aligned}$$

18 $$\begin{aligned} V_{R_0}&=I \ {R_0},\quad \text {and} \end{aligned}$$

19 $$\begin{aligned} \dot{V_c}&=\frac{I}{C_c}, \end{aligned}$$

where $V_{batt}$ can be directly correlated to the resulting electromotive torque considering the armature resistance and inertia of the motors as

20 $$\begin{aligned} V_{batt}&=L_a \ \frac{dI}{dt} +{R_a}{I}+{K_b} \ {\omega },\end{aligned}$$

21 $$\begin{aligned} T_{mot}&= T_{load}+J\dot{\omega } +b\omega , \quad \text {and}\end{aligned}$$

22 $$\begin{aligned} T_{mot}&=K_t \ {I}. \end{aligned}$$

A summary of all modeling parameters and respective numerical values are given in Table 2. Experimental datasets have been gathered during multiple dynamometer testing procedures of the electric vehicle under study, which have been used for iterative estimation and tuning of the model parameters as explained in the sequel.

**Table 2:** Electric vehicle modeling parameters.

Vehicle Parameters^36^				Tire Modeling Parameters^37^			
Symbol	Description	Value	Unit	Symbol	Description	Value	Unit
*W*	Gross vehicle weight	2500	$\mathrm {[N]}$	*B*	Stiffness factor	0.178	$\mathrm {[-]}$
*F*	Frontal projected area	2.195	$\mathrm {[m^2]}$	*C*	Shape factor	1.55	$\mathrm {[-]}$
*K*	Air drag coefficient	0.38	$\mathrm {[N \cdot s^2/m^4]}$	*D*	Peak factor	2193	$\mathrm {[-]}$
*f*	Coefficient of rolling resistance	0.02	$\mathrm {[-]}$	*E*	Curvature factor	0.432	$\mathrm {[-]}$
$l_f$	Center of gravity to front axle distance	1108	$\mathrm {[mm]}$	$h_{\textrm{cg}}$	Center of gravity height	0.65	$\mathrm {[m]}$
$l_r$	Center of gravity to rear axle distance	562	$\mathrm {[mm]}$	$r_d$	Dynamic radius of tire	0.3059	$\mathrm {[m]}$
*l*	Vehicle wheelbase	1670	$\mathrm {[mm]}$				

Motor Parameters to be Estimated^16^				Battery Parameters to be Estimated^16^			
Symbol	Description	Value	Unit	Symbol	Description	Value	Unit
*J*	Moment of inertia	–	$\mathrm {[kg \cdot m^2]}$	$V_{\textrm{oc}}$	Open circuit voltage	–	$\mathrm {[V]}$
$K_t$	Torque constant	–	$\mathrm {[N \cdot m/A]}$	$R_o$	Ohmic resistance	–	$\mathrm {[\Omega ]}$
*b*	Bearing damping coefficient	–	$\mathrm {[N \cdot s/m]}$	$C_c$	Series capacitance	–	$\mathrm {[F]}$
$K_b$	Back-EMF constant	–	$\mathrm {[V \cdot s/\textrm{rad}]}$	$R_b$	Polarization resistance	–	$\mathrm {[\Omega ]}$
$R_a$	Armature resistance	–	$\mathrm {[\Omega ]}$	$C_b$	Polarization capacitance	–	$\mathrm {[F]}$
$L_a$	Armature inductance	–	$\mathrm {[H]}$				

## Adaptive cruise control using hybrid-mode MPC

This section presents a novel MPC formulation for ACC system design. Instead of using independent MPCs for each mode, the proposed approach employs a hybrid framework to reduce the computational burdens and redundancy. Thus, a unified prediction model is employed across ACC modes, utilizing different weighting matrices and reference signals dedicated to each mode. This approach targets the control objectives of each mode with minimal computational effort.

The proposed cruise control system aims to handle critical self-driving situations, classified based on inter-vehicle distance and relative speed to the lead vehicle. To this aim, the main control scheme comprises three operating modes, namely: cruising, spacing, and braking. In the first mode, retention of a given speed is the main control objective, while the main difference between spacing and braking mode (Coasting Performance) is crossing the threshold of minimum safe inter-distance^38^.

**Fig. 3:**
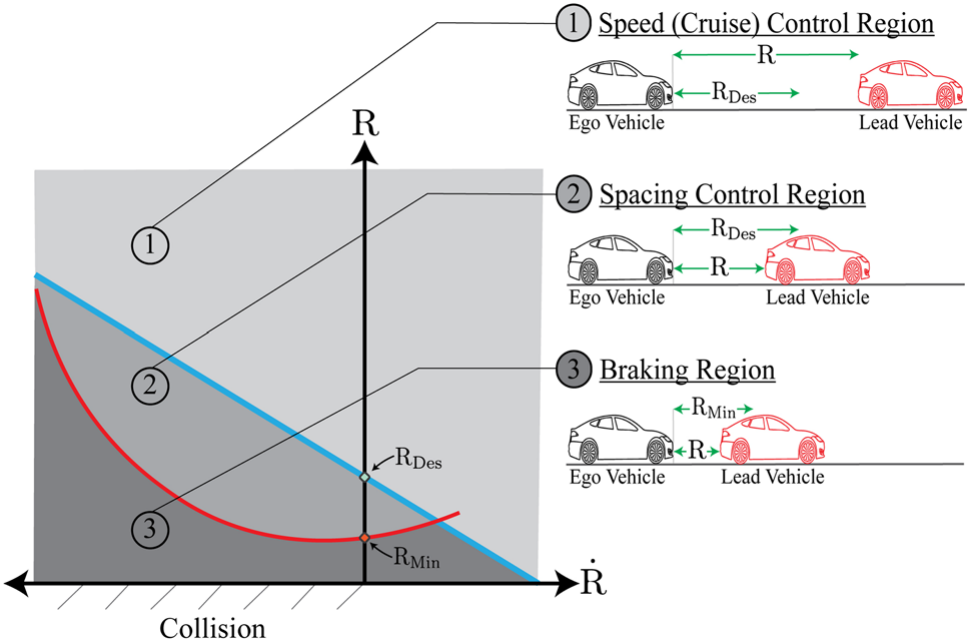
Typical relationship between range and range rate for a vehicle equipped with ACC system under different driving scenarios.

Swapping between the operating modes is conducted through a hybrid-mode MPC with unified state variables that operate across three distinct control regions based on received LiDAR measurements. The classification of regions to which control modes are assigned is illustrated in Fig. 3 based on the relationship between range measurements (*R*) relative speed $(\dot{R})$.

It is required for the hybrid MPC to conduct adequate control actions according to the circumstances of ego vehicle in each region and any encountering vehicles and prospective maneuvers. In region 1, it is required to maintain a predefined speed, while -as can be perceived from Fig. 3– that the collision risk is minimal. In this region, the controller only operates in cruise or headway mode based on the comparison of the instantaneous distance (*R*) relative to ($R_{Des}$).

In Region 2, the inter-distance to surrounding vehicles increases according to the lower parabolic borderline, which implies course of actions to retain constant separation distance at steady-state. The boundary line between cruising and headway control can be defined as

23 $$\begin{aligned} {R}&=-{T} \dot{{R}}+{R_{Des}}, \end{aligned}$$

where (*T*) is the slope of the transitioning line and represents the time constant of the vehicle longitudinal dynamics, and ${(R_{Des})}$ is the desired inter-vehicle distance defined as

24 $$\begin{aligned} {R_{Des}}&={h}{{v_{L}}}+{L}, \end{aligned}$$

where (*h*), $(v_{L})$, and (*L*) denote the constant headway time parameter, lead vehicle velocity, and standstill separation distance, respectively.

**Fig. 4:**
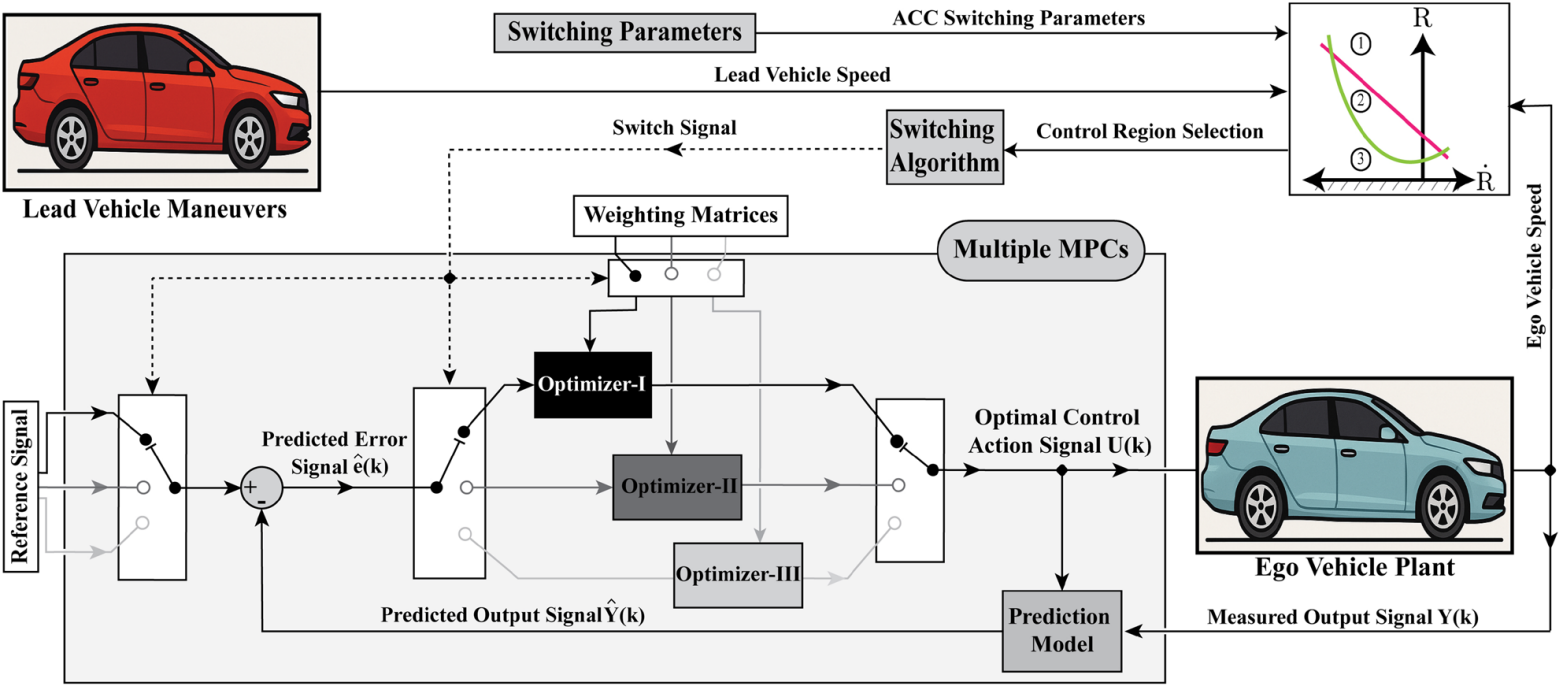
Structure of the proposed switched MPC control algorithm.

The parabolic boundary line between regions 1 and 2 is defined as

25 $$\begin{aligned} {R}&={R_{Min}}+\frac{(\dot{{R}})^2}{{2 D_{max}}}, \end{aligned}$$

where $(R_{Min})$ is the minimum permissible distance and $(D_{max})$ is the maximum Ego vehicle’s deceleration. The headway transition trajectory equation contains the term ($(\dot{{R}})^2$), which makes the parabola symmetric about the vertical axis and hence the practical implementation of the control strategy is inherently asymmetric. Crossing the minimum inter-distance in region 3 immediately activates the braking mode (Coasting Performance) to mitigate the risk of rear-end collisions. The integration of the proposed control scheme into the upper level architecture is illustrated in Fig. 4.

This study employs a simplified linear prediction model with the primary objective of evaluating the proposed hybrid-mode control architecture, rather than being dominated by the complexity of a nonlinear prediction model. Incorporating a nonlinear MPC formulation, together with a state observer, would impose a substantial computational burden and could potentially obscure the evaluation of the proposed approach, which is mainly focused on unifying this simplified prediction model within the MPC framework alongside ACC mode-switching capability.

Mathematical representation of the proposed hybrid MPC can be formulated based on the traction dynamics defined in (20-22) and operation modes in (23-25) as

26 $$\begin{aligned} \begin{bmatrix} a_{e} \\ \dot{R} \end{bmatrix}&= \begin{bmatrix} \frac{-b}{r_d \sigma _{2}} & 0 \\ -1 & 0 \end{bmatrix} \begin{bmatrix} v_{e} \\ R \end{bmatrix} + \begin{bmatrix} \frac{\sigma _{1}}{\sigma _{2}} & 0 \\ 0 & 1 \end{bmatrix} \begin{bmatrix} \tau \\ v_{L} \end{bmatrix}, \end{aligned}$$

where $(\tau )$ is the throttle voltage signal, $(v_{L})$ the lead vehicle speed and the output vector is derived identically to the state vector. The coefficients $(\sigma _{1})$ and $(\sigma _{2})$ are defined as

27 $$\begin{aligned} \sigma _{1}&=\frac{K_{t}\,\mathrm (I)^{max}-\frac{K\,F\, r_{d}(\mathrm ({v_{e}^{max}))}^2}{2}}{\mathrm (\tau ^{max})-\mathrm (\tau ^{min})} \quad \text {and} \end{aligned}$$

28 $$\begin{aligned} \sigma _{2}&=\frac{J}{r_{d}}+\frac{W \times r_{d}}{g}, \end{aligned}$$

where the superscripts $.^{max}$ or $.^{min}$ denote the maxima and minima of relevant variables, which have been defined experimentally during dynamometer testing. The established prediction model for vehicle speed anticipation is the same for all modes of the proposed MPC.

Based on predicted values of $v_e$, the estimated error, i.e. deviation from reference value, is calculated and a sequence of control actions is generated online within a constrained optimization problem defined as

29 $$\begin{aligned} \min _{u_{0},...,u_{k+N_{c}-1}} J(k), \end{aligned}$$

such that

30 $$\begin{aligned} {\begin{matrix} J(k) & = \sum _{i=0}^{N_{p}} \left[ \hat{y}(k+i|k) - r(k+i|k)\right] ^T .\boldsymbol{Q}.\left[ \hat{y}(k+i|k) - r(k+i|k)\right] + \sum _{i=0}^{N_{p}} u^T(k+i|k).\boldsymbol{N}.u(k+i|k) \\ & \quad + \sum _{i=0}^{N_{c}-1} \left[ \Delta u^T(k+i|k).\boldsymbol{R}.\Delta u(k+i|k)\right] , \end{matrix}} \end{aligned}$$

subject to

31 $$\begin{aligned} u_{min} \le u(k+i) \le u_{max}, \quad \quad&i=0,\ldots ,N_{p}-1 \end{aligned}$$

32 $$\begin{aligned} y_{min} \le y(k+i) \le y_{max}, \quad \quad&i=1,\ldots ,N_{p}-1 \end{aligned}$$

33 $$\begin{aligned} \Delta u_{min} \le \Delta u(k+i) \le \Delta u_{max}, \quad \quad&i=0,\ldots ,N_{c}-1 \end{aligned}$$

where a unified prediction and control horizons $N_{p}$ and $N_{c}$ of 10 s and 3 s respectively have been considered for all hybrid modes of the MPC. The weighting matrices ($\boldsymbol{Q}$), ($\boldsymbol{N}$), and ($\boldsymbol{R}$) denote the output error weight matrix, the control action weight matrix, the weight matrix of the control action’s rate of change, respectively. A summary of the control algorithm parameters in the different operating modes is given in Table 3.

**Table 3:** Control parameters and reference signals.

Parameter	Cruising	Headway	Braking	Unit	Parameter	Cruising	Headway	Braking	Unit
*Horizon Parameters*					*Actuator Constraints*				
Prediction horizon ($N_p$)	10	10	10	steps	Min throttle voltage ($u_{\min }$)	1.2	1.2	0	V
Control horizon ($N_c$)	3	3	3	steps	Max throttle voltage ($u_{\max }$)	4.9	4.9	4.9	V
Sampling time ($T_s$)	0.1	0.1	0.1	s	Max throttle rate ($\Delta u_{\max }$)	0.5	0.5	1.0	V/s
*Output Weighting Factors*					*Safety Constraints*				
Velocity error weight ($Q_{11}$)	100	50	10	—	Min velocity ($v_{\min }$)	0	0	0	m/s
Distance error weight ($Q_{22}$)	0	100	500	—	Min separation ($R_{\min }$)	—	3.0	3.0	m
*Control Signal Weighting Factors*					*Reference Signals*				
Control effort weight (*N*)	1	1	0.1	—	Velocity reference ($r_v$)	Driver set	$v_L$	0	m/s
Control rate weight (*R*)	10	10	5	—	Distance reference ($r_R$)	—	$R_{\text {Des}}$	$R_{\text {Min}}$	m

## Experimental testing procedures and offline model-parameter estimation

The validation of the proposed control algorithm is a milestone of this contribution, which addresses the put forth gap in literature of real-time ACC implementations^39,40^. The experimental testing procedures have been conducted on an EV–prototype with design specifications explained previously in^36^. To this aim, the testing procedures of the proposed method have been conducted both indoors (using a chassis dynamometer) and outdoors (as a real road test).

During each test, Offline estimation of model parameters and experimental application of the proposed ACC based on hybrid-mode MPC are implemented simultaneously. Using offline parameter estimation allows clear evaluation of the proposed control method, which is the main focus of this study, without adding extra computational cost to the MPC from real-time parameter estimation.

**Fig. 5:**
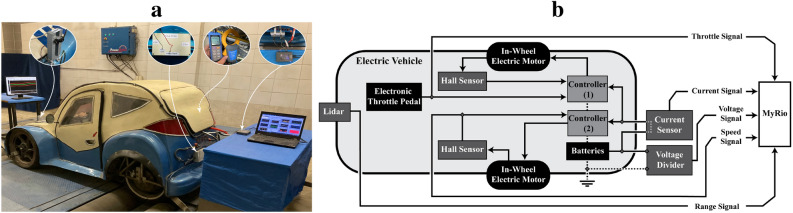
Experimental setup of test vehicle with on-board data acquisition system on the chassis-dynamometer. **a**, Vehicle setup with integrated modules. **b**, Layout diagram of attached sensors.

The estimation of depicted modeling parameters requires a dataset of real measurements for both identification and validation. Thus, the test vehicle has been equipped with an onboard data acquisition system during standardized chassis-dynamometer driving tests, as illustrated in Fig. 5a. A unified platform (myRIO-1900) is implemented to host the depicted model/control algorithms and to acquire all measurement and control signal.

The on-board measurement system includes a Hall-effect sensor (electric motor-integrated), a current sensor, and a designed voltage divider to measure the vehicle speed, battery current, and battery voltage, respectively. The electronic throttle pedal voltage was measured using a cut-in signal technique. Furthermore, a front-mounted LiDAR is implemented to measure the inter-vehicle distances during tests. The schematic layout in Fig. 5b illustrates the measured signals and the attached sensors.

During chassis-dynamometer testing, experiments have been conducted at down-scaled speeds as braking commands of the vehicle are strictly prohibited. Accordingly, tracing the speed profile of the test driving cycle has been conducted by inertial–coasting for deceleration. Taking that into consideration, the control outputs are adaptively calculated and analyzed to validate the efficacy of the proposed ACC module under real-time disturbances.

The gathered measurements are transferred to MATLAB/Simulink environment to be used as reference datasets for parameters estimation. The Offline estimation algorithm has been initially introduced by the authors in^16^. It depends on the trust-region reflective algorithm, which tunes modeling parameters, either for battery or electric motor, using a least-squares technique to achieve convergence with the reference signals.

**Fig. 6:**
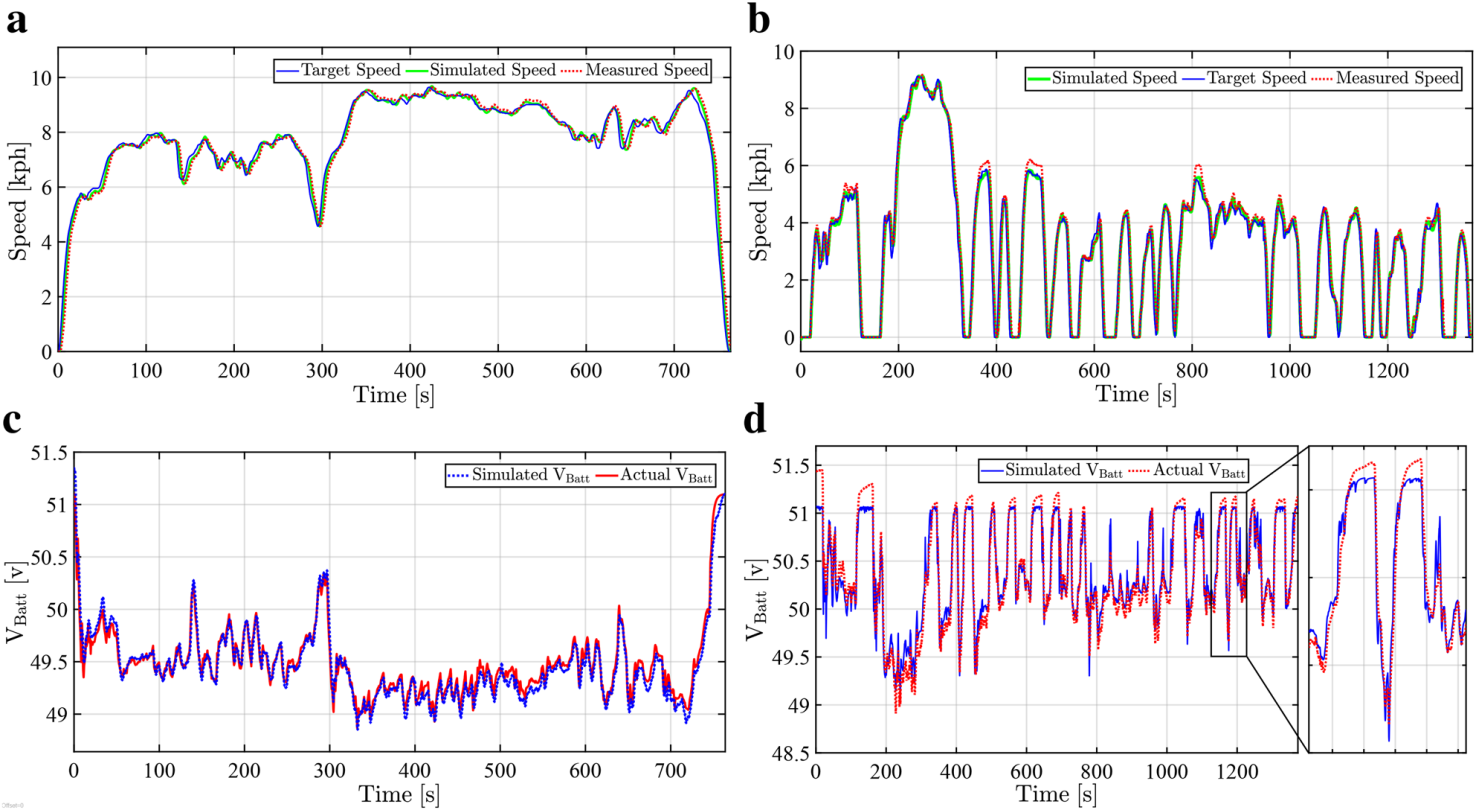
Experimental validation of the developed electric vehicle powertrain model under two standardized driving cycles. **a**, Comparison between the reference and actual motor speed profiles under the scaled highway driving cycle. **b**, Comparison between the reference and actual motor speed profiles under the scaled urban driving cycle. **c**, Battery terminal voltage response under the scaled highway driving cycle. **d**, Battery terminal voltage response under the scaled urban driving cycle.

The estimation of modeling parameters for the electric battery and vehicle dynamics are conducted through various driving cycles. Fig. 6 illustrates the Highway and UDDS cycle validation results, confirming the model’s robustness across diverse operating conditions before ACC controller implementation. The acquired parameters of the battery demonstrate a high degree of consistency between the actual measurements and the simulation, as shown in Figures 6c and 6d for highway and urban cycles, respectively. Furthermore, accurate estimation results of electric motor and vehicle dynamics parameters led to a good match between the actual and simulated speed profiles, as shown in Figures 6a and 6b for highway and urban cycles, respectively.

To quantitatively evaluate the model validation, Table 4 summarizes the numerical error measures across three different driving cycles. The Root Mean Square (RMS) error between measured and simulated data was calculated for vehicle speed and battery voltage during traction. The results demonstrate an RMS error of less than 3 [km/h] and 0.4 [V], respectively.

**Table 4:** Validation of the proposed models’ simulated results with actual vehicle data according to different driving cycles.

	Root mean square error		
	Highway	Urban	Japanese-10
Vehicle speed [m/s]	0.2788	0.4044	0.7614
Battery voltage [v]	0.0855	0.1357	0.3786

**Fig. 7:**
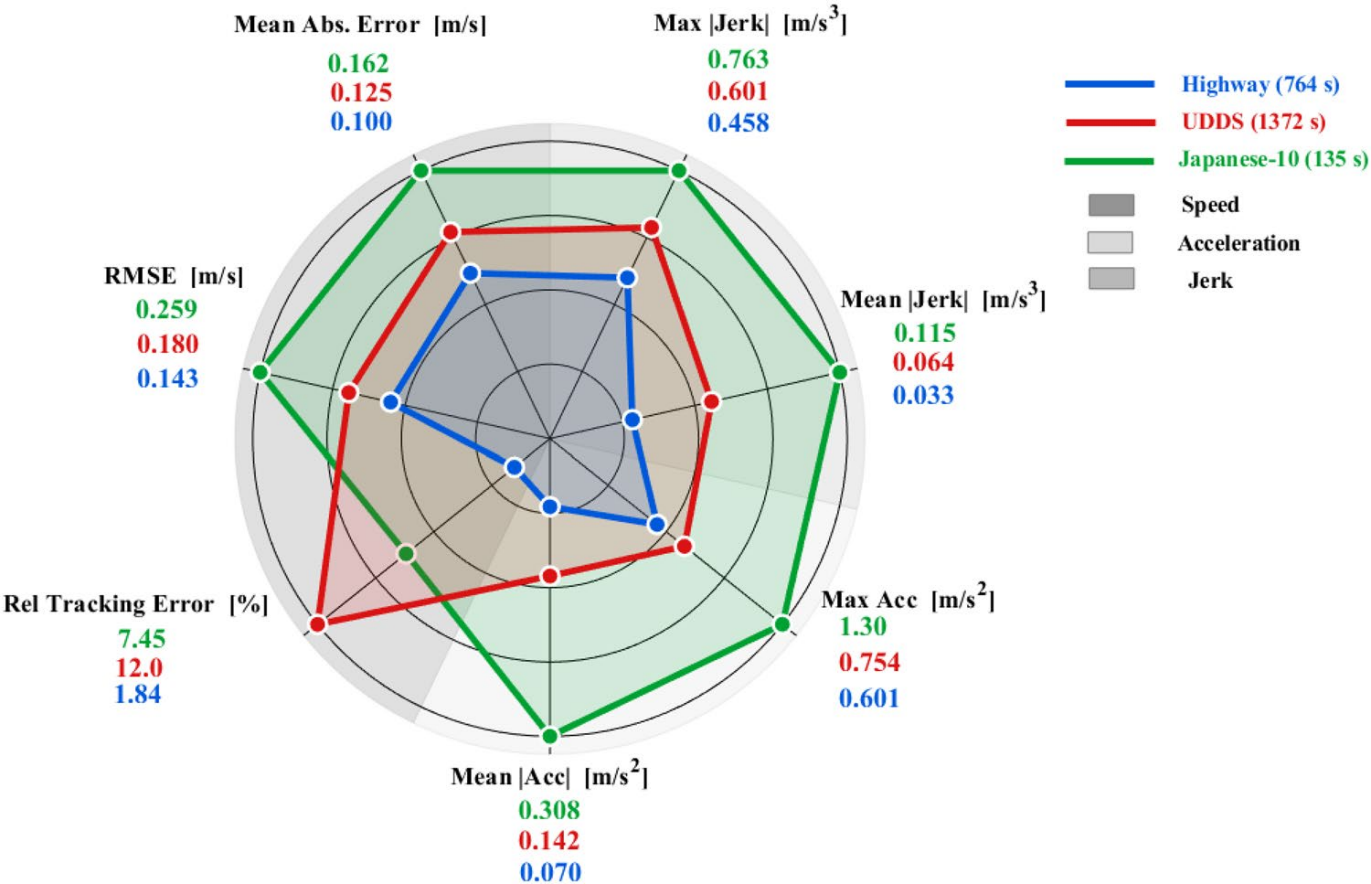
Implicit driver behavior characterization during tracing different driving cycles.

To implicitly characterize the aggressiveness diversity of the driving cycles used for model estimation and validation, a statistical analysis is presented in Fig. 7. Seven performance and dynamic metrics are grouped into velocity tracking, acceleration, and jerk categories. They are evaluated across the three cycles. The results reveal that the Japanese-10 cycle reflects aggressive driving behavior, the Highway cycle represents conservative driving, and the UDDS cycle shows a moderate profile. Comprehensive human-in-the-loop validation with classified driver profiles is recognized as a limitation and is identified as a future research direction.

**Fig. 8:**
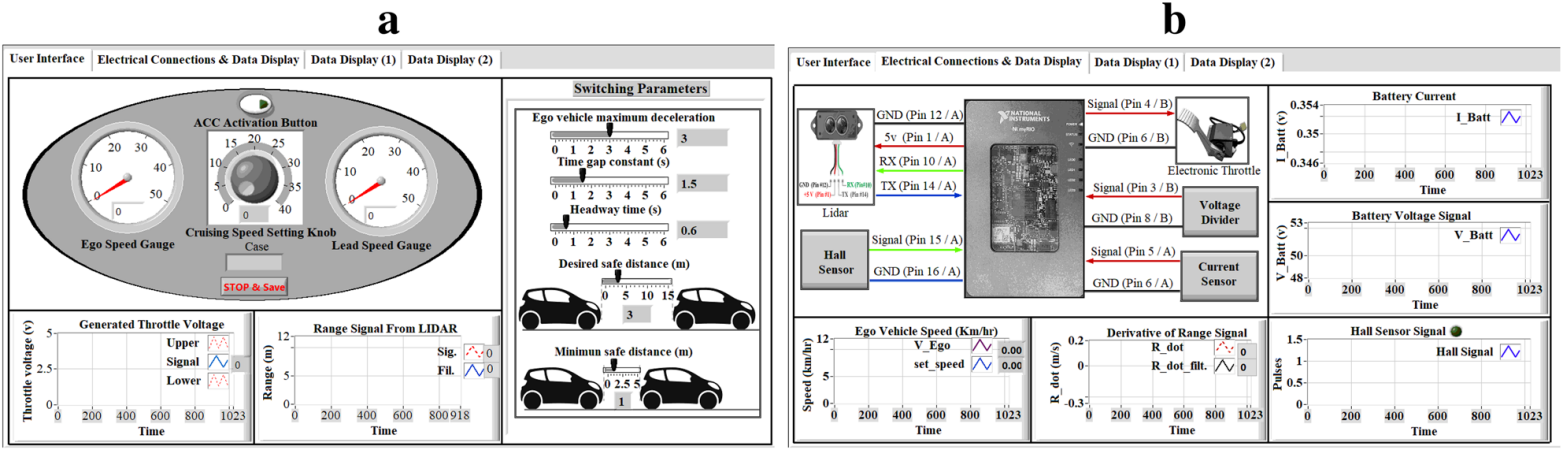
The proposed user interface for entering ACC system parameters, recording measured signals, and displaying all data. **a**, The user interface of the proposed ACC system. **b**, Electrical connections to MyRio for real-time implementation.

Following successful parameter tuning, the validated model is integrated into the unified prediction module for the hybrid-mode MPC. This integration underscores the necessity of high-fidelity modeling for achieving the control objectives established in the mathematical modeling section. To facilitate real-time data monitoring and the adjustment of ACC parameters during laboratory experiments, a dedicated Graphical User Interface (GUI) was developed as shown in Fig. 8.

Through the dedicated GUI, the user is enabled to change marginal boundaries of the intra-distance, maximum deceleration, time gap constant, and headway time. Besides, real-time values of front/ego vehicle speeds, LiDAR signals, and throttle control signals are depicted. The right window illustrates the main connections and signals’ plots.

The execution time of the control while loop in LabVIEW was monitored on the NI myRIO-1900 (ARM Cortex-A9, 667 MHz), yielding an average computation time of approximately 12 ms per cycle to solve the quadratic programming (QP) and compute the control action. This represents only 12% of the 100 ms sampling period, leaving sufficient margin for sensor data acquisition, mode switching logic, and communication overhead. No latency occurred during real-time deployment, confirming the feasibility of the approach on resource-limited embedded hardware. Detailed per-cycle timing monitoring across varying operating conditions is planned for future work to characterize the real-time performance further.

## Results analysis

**Fig. 9:**
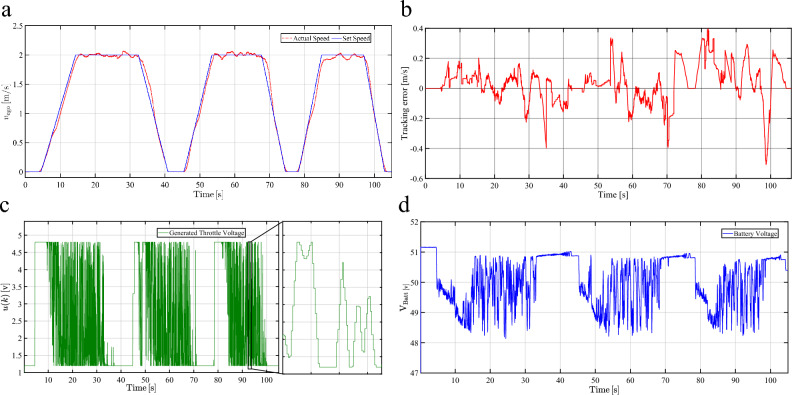
Experimental results of the cruise control mode for the MPC-based ACC system. **a**, Comparison between the vehicle’s actual speed and the proposed target speed of the cruising control mode. **b**, Speed tracking error during the cruise control mode. **c**, Control action signal generated from myRIO. **d**, Battery terminal voltage response during the cruise control mode.

The achievable performance of the proposed ACC is evaluated considering real-time applicability and the ability to conduct different challenging maneuvers during cruising, spacing, and braking. The first control mode, cruising control, aims to maintain the vehicle speed constant. An arbitrary speed profile is set as the reference speed to evaluate the vehicle response in tracking the speed profile as shown in Fig. 9.

Fig. 9a demonstrates a high degree of consistency between the arbitrary speed profile and the actual vehicle’s speed. The error between the actual and set speed profiles is shown in Fig. 9b, which confirms the controller’s high performance in tracking the driver’s set speed. The throttle control signal is calculated based on the anticipated model response as illustrated in Fig. 9c. The battery terminal voltage response over the cycle is also represented in Fig. 9d. The controller’s generated signal continuously adjusts the electric vehicle’s speed to ensure proper tracking of the reference speed. The second tested mode is the spacing control, in which the ACC attempts to maintain a predetermined safe distance to the preceding vehicle. For optimal headway control, the MPC considers the speed of the front vehicle as a reference signal, while maintaining a safe separate distance between them in the steady-state.

The third tested mode is the braking mode (Coasting Performance), in which the ACC conducts incremental speed reduction when approaching the lead with a distance less than the minimum permissible inter-vehicle distance (here considered as 3 meters). The minimum separation distance was chosen to exceed the typical standstill gap^41^, which provides a safe margin to tackle potential measurement errors and control delays. This ensures that the actual gap stays above the collision-free boundary.

**Fig. 10:**
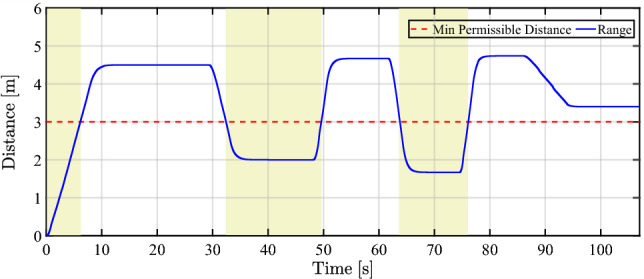
The proposed arbitrary range profile to evaluate the ACC system’s spacing (white regions) and braking (yellow regions) modes.

The experimental evaluation of the proposed ACC in the spacing mode is implemented using a chassis dynamometer. To this aim, an arbitrary range profile is considered instead of utilizing the LiDAR measurements. The range profile, which is represented in Fig. 10, reflects the change in inter-vehicle distance to emulate the exposure to an approaching lead vehicle. This arbitrary range profile emulates realistic urban lead vehicle maneuvers measured by LiDAR sensors. High-slope range profile (0.5–1.0 m/s) replicates sudden braking or range closure by the lead vehicle, followed by constant values to represent steady-state car-following at safe inter-vehicle distances typical of urban traffic. A minimum of 3 m threshold crossings are defined to trigger critical mode-switching events, and repeated cycles reflect stop-go traffic dynamics. This profile features high-slope segments between spacing and braking mode (coasting performance) to validate transient mode-switching, followed by constant values to evaluate the steady-state response of the controller.

**Fig. 11:**
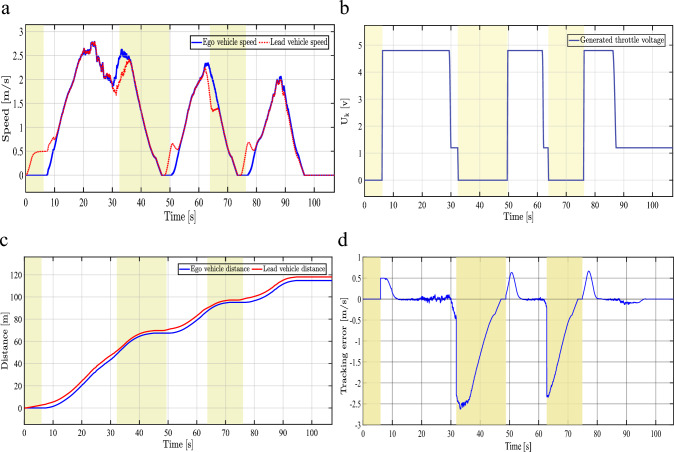
Experimental results during spacing and braking control modes, demonstrating the MPC-based ACC system’s ability to maintain a safe inter-vehicle distance. **a**, Speed profiles of the ego and lead vehicles under braking mode (yellow regions) and spacing mode (white regions). **b**, Throttle voltage signal generated by the myRIO controller based on MPC computations during braking and spacing modes. **c**, Total distance traveled by both vehicles over the driving cycle. **d**, Velocity tracking error, with spacing mode (white regions) and braking mode (yellow regions) highlighted.

Figure 11 illustrates the achieved results in two different modes, namely: spacing (white regions) and braking mode (Coasting Performance) (yellow regions). In braking mode (Coasting Performance), the controller attempts to stop the ego vehicle as the lead vehicle stops. It can be perceived from Fig. 11b that the generated throttle voltage signal from myRIO is zero in the braking regions, where the vehicle is decelerated by its inertia only. The controller adapts the required time gap constants for this limitation during dynamometer testing, which is not the typical case during road-tests as discussed in the sequel.

As the intra-distance to the lead vehicle increases, the control mode switches back to spacing. Consequently, the MPC switches its operating point from braking the ego vehicle to driving at the same speed of the lead vehicle as shown in the white regions of Fig. 11a. The controller generates the maximum control action signal of the throttle pedal (4.8 [V]), as shown in the white regions of Fig. 11b. The total distance traveled by the ego vehicle and the lead vehicle, in Fig. 11c, reveals the high similarity in the distance profile for both vehicles. Fig. 11d shows the spacing and braking mode tracking error. During spacing mode (white regions), the RMSE of speed difference between the ego and lead vehicles is 0.1973 m/s. The yellow-shaded regions indicate braking mode, where the reference switches to zero velocity. The larger deviations observed during braking (up to $-2.5$ m/s) are attributed to the coasting-only deceleration constraint.

**Fig. 12:**
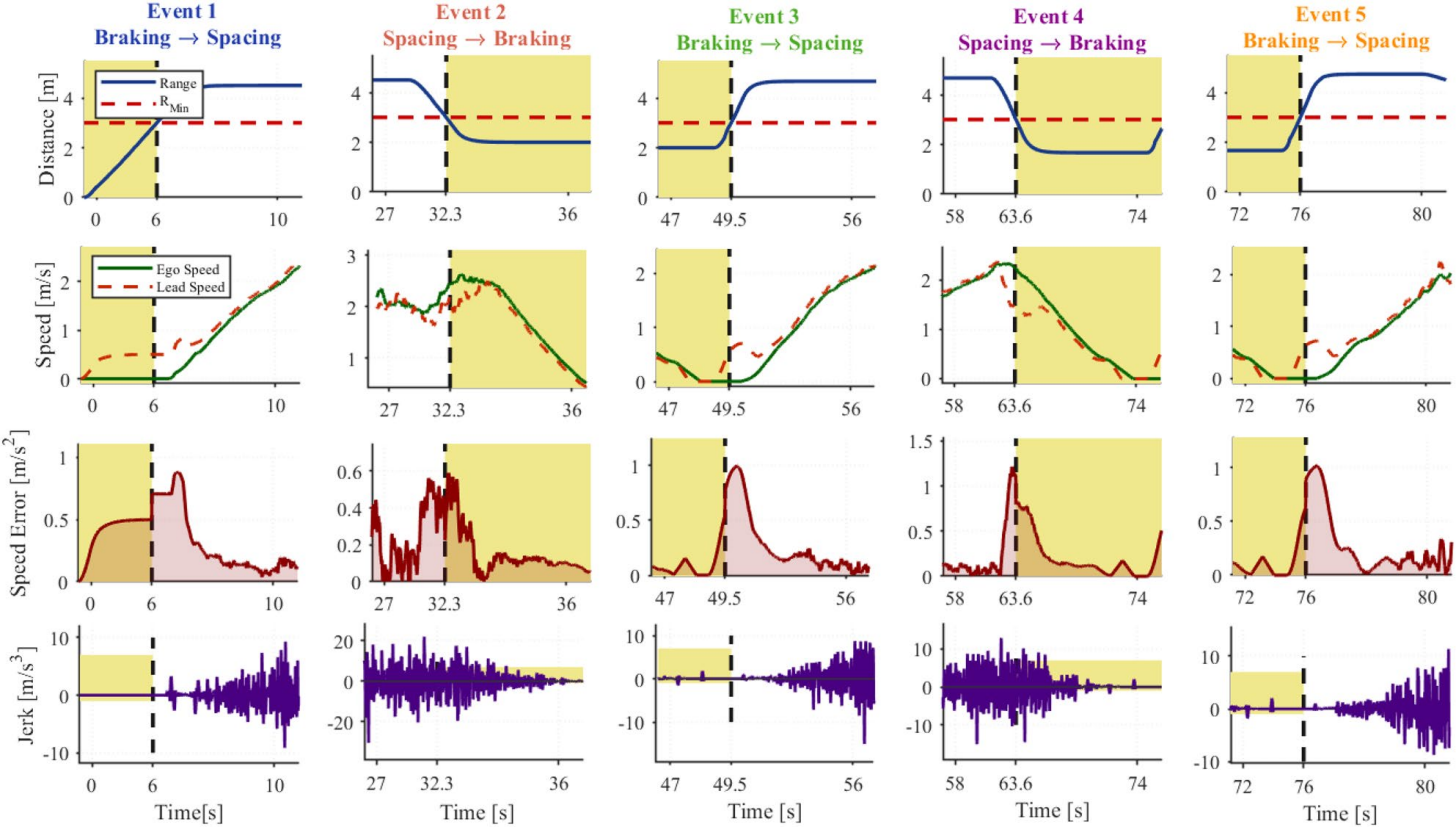
Transient response characteristics at ACC mode transition points across five switching events.

To further validate bounded switching behavior, The Fig. 12 isolates five representative switching events and presents four signals (inter-vehicle distance, speed tracking, speed error, and jerk), accompanied by a new quantitative summary Table 5, reporting settling time, rise/fall time, maximum speed error, RMSE, maximum jerk, minimum Time To Collision (TTC), and minimum range for each event.

The results demonstrate that transitions towards spacing mode have an average settling time of 1.67 s, a RMSE of 0.285 m/s, and an infinite TTC, with a minimum range of 3.004 m, indicating smooth and safe transitions. Transitions from spacing to braking modes, which are inherently more demanding, yield an average settling time of 1.80 s, RMSE of 1.479 m/s, minimum TTC of 3.48 s, and minimum range of 1.835 m, all within safe operational bounds. Across all events, the speed error converges within approximately 1.7–1.8 s, jerk remains within physically acceptable limits, and safety-critical constraints are never violated.

**Table 5:** Transient response performance metrics of the ACC system at mode transition points.

Unit	Settling time [s]	Rise/Fall Time [s]	Max. Speed Error [m/s]	RMSE of Speed [m/s]	Max. Jerk [m/s^3^]	Min. TTC [s]	R_min_ [m]
**Event 1**	2.00	9.55	0.622	0.284	9.288	$\infty$	3.000
**Event 3**	1.50	9.18	0.704	0.283	15.051	$\infty$	3.003
**Event 5**	1.50	7.97	0.720	0.287	11.386	$\infty$	3.009
**Average**	**1.67**	**8.90**	**0.682**	**0.285**	**11.909**	$\boldsymbol{\infty }$	**3.004**
**Event 2**	1.20	8.76	2.628	1.849	19.558	4.26	2.001
**Event 4**	2.40	7.47	2.235	1.108	14.526	2.70	1.670
**Average**	**1.80**	**8.11**	**2.431**	**1.479**	**17.042**	**3.48**	**1.835**

**Fig. 13:**
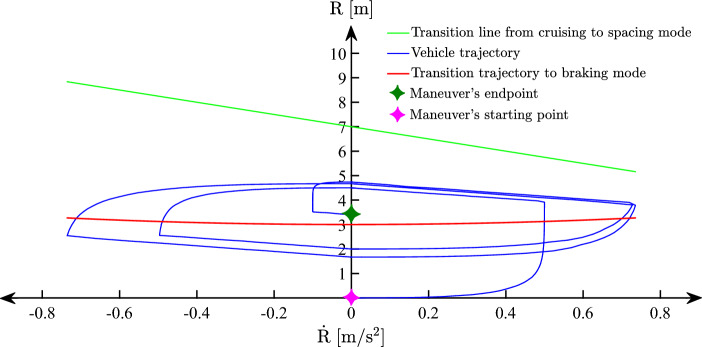
The relationship between the vehicle trajectory and the transition trajectories on the range vs. range rate diagram.

The overall maneuver trajectory is represented in the *R*–$\dot{R}$ diagram in Fig. 13, which is a typical perspective to evaluate the performance of ACC systems. Considering the switching parameters identified in equations 23 and 25, the ACC switching algorithm creates a headway transition line and a braking transition trajectory to distinguish between different control modes. As shown in Fig. 13, the vehicle trajectory (blue line) is circulating without crossing the horizontal axis line until it reaches the final (desired) steady-state range.

Quantitative evaluation measures of the above-explained results are summarized in Table 6. In cruise control, the driver-defined set speed is compared with the electric vehicle’s actual speed. While spacing control mode, the speed of the ego vehicle is compared with the lead vehicle’s speed. In braking mode (Coasting Performance), RMSE is calculated by comparing the ego vehicle speed with zero velocity.

It can be perceived that the speed error (represented as RMS) in both cruise and headway control has been retained minimal ($<0.2 [m/s]$). However, this value has been slightly higher in braking mode (Coasting Performance) on dynamometer conditions, as the vehicle decelerates by coasting in this mode, i.e. no braking commands are executed in this case. This puts forward the necessity to conduct on-road testing to investigate the ACC performance further.

**Fig. 14:**
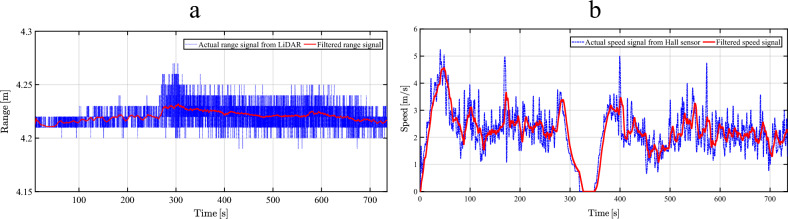
Sensor signal filtering for raw LiDAR range and Hall sensor speed measurements. **a**, Comparison between the raw LiDAR range signal and the filtered range signal over the driving cycle. **b**, Comparison between the raw Hall sensor speed signal and the filtered speed signal over the driving cycle.

An extended drive test has been conducted under realistic road conditions, yet with inertial coasting substituting braking commands, to ensure the functional efficacy of the controller. For safety concerns, the allowed reference speed for autonomous driving on road testing has been scaled-down to 18 [km/h]. The implemented test scenario comprises repeated cycles of acceleration/deceleration and a sudden-stop of the front vehicle. To address range-sensor imperfections in outdoor experiments, raw LiDAR measurements are filtered with a low-pass filter before range-rate computation via differentiation. This eliminates pulsations and odd spikes that don’t reflect true vehicle behavior. As demonstrated in Fig. 14a, the filtered range signal closely tracks measurements without high-frequency ripples. Consequently, Fig. 14b shows the corresponding vehicle speed signals, confirming near-elimination of derivation-induced noise spikes that could cause illogical lead vehicle speed estimates.

**Fig. 15:**
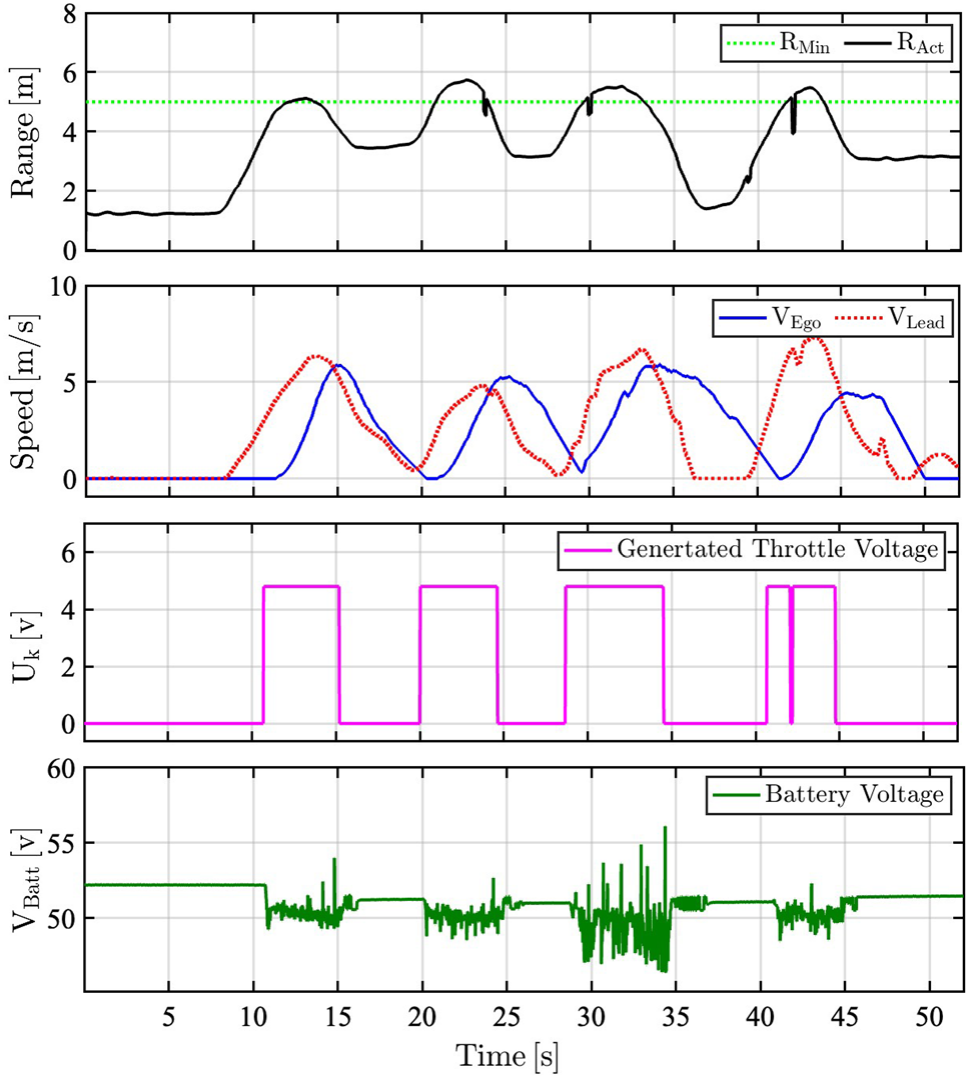
Experimental on-road testing procedures.

It can be perceived from the achieved results of road-testing in Fig. 15 that the average intra-distance (*R*= 5 [m]) has been successfully retained during cruising and spacing modes with an average deviation less than 1.7 [m]. The sudden stop of the front vehicle has been robustly handled with inertial coasting, sustaining a minimum distance of 1.8 [m]. The overall performance of the proposed controller has proved a significant potential to handle the mechanical limitations of the experimental vehicle and the inherent measurement uncertainty of installed low-cost sensors during real road-testing.

**Table 6:** RMSE of ego vehicle speed computed for each control mode of the system.

	Root mean square error of ego vehicle speed [m/s]		
	Cruise control mode	Headway control mode	Braking mode (Coasting Performance)
**Compared to**	Driver’s set speed	Lead vehicle speed	zero velocity
**RMSE [m/s]**	0.0883	0.1973	0.7243

## Conclusion

This study proposes a novel hybrid-mode MPC for ACC that is capable of handling complex events and driving scenarios. The computational requirements of the proposed method are mitigated through a unified prediction module reliant on LiDAR measurements to suit realtime applicability. Experimental validation has been conducted via chassis-dynamometer and on-road testing.

The hybrid-mode MPC for an ACC system was developed, applied, and evaluated for an electric vehicle. A high-fidelity model of an all-electric experimental vehicle was implemented, including vehicle longitudinal dynamics, the traction E-motor, and the battery model. Offline system-parameters identification of the electric motor and the battery was conducted during hardware-in-the-loop testing. A three-phase testing procedures have been carried out, starting with an experimental model validation test on a chassis dynamometer.

The ensuing testing phase comprised an autonomous drive test using the proposed hybrid MPC-based ACC system for an in-house driving cycle test and a real on-road test, respectively. The proposed control algorithm proved its robustness in the standard cruise control mode operation. Additionally, the transitions between the cruise control, headway control, and braking operating regions were smooth and stable. The RMSE between the desired values and the actual values of the vehicle speed in the cruise control, headway control, and braking (Coasting Performance) modes were 0.088, 0.197, and 0.72, respectively.

Although the proposed control strategy yielded promising results, several limitations should be considered, including the reliance on inertial coasting braking during chassis dynamometer testing, a comprehensive comparison with other control methodologies, offline parameter estimation integrated in a linearized prediction model within the MPC framework, and a comprehensive sensitivity analysis of the estimated parameters. These limitations induce a loss of prediction accuracy in the MPC, particularly when system modeling parameters deviate due to disturbances or long-term deterioration of EV subsystems. Additionally, relying on coasting braking increases collision risks, especially during high-speed maneuvers.

The proposed work paves the way for multiple research improvements, including evaluation of the string stability and highway testing with an advanced brake system. Thus, future research aims to further enhance the proposed approach through integration of active braking mechanisms, evaluation of string stability, highway testing, and comprehensive human-in-the-loop studies.

## Data Availability

The datasets used and/or analyzed during the current study available from the corresponding author on reasonable request.
